# Findings from Studies Are Congruent with Obesity Having a Viral Origin, but What about Obesity-Related NAFLD?

**DOI:** 10.3390/v13071285

**Published:** 2021-07-01

**Authors:** Giovanni Tarantino, Vincenzo Citro, Mauro Cataldi

**Affiliations:** 1 Department of Clinical Medicine and Surgery, “Federico II” University Medical School of Naples, 80131 Napoli, Italy; 2 Department of General Medicine, “Umberto I” Hospital, Nocera Inferiore (Sa), 84014 Nocera Inferiore, Italy; v.citro@libero.it; 3 Section of Pharmacology, Department of Neuroscience, Reproductive Sciences and Dentistry, “Federico II” University of Naples, 80131 Napoli, Italy; cataldi@unina.it

**Keywords:** adenovirus, obesity, NAFLD, viral persistence, chronic low-grade inflammation

## Abstract

Infection has recently started receiving greater attention as an unusual causative/inducing factor of obesity. Indeed, the biological plausibility of infectobesity includes direct roles of some viruses to reprogram host metabolism toward a more lipogenic and adipogenic status. Furthermore, the probability that humans may exchange microbiota components (virome/virobiota) points out that the altered response of IFN and other cytokines, which surfaces as a central mechanism for adipogenesis and obesity-associated immune suppression, is due to the fact that gut microbiota uphold intrinsic IFN signaling. Last but not least, the adaptation of both host immune and metabolic system under persistent viral infections play a central role in these phenomena. We hereby discuss the possible link between adenovirus and obesity-related nonalcoholic fatty liver disease (NAFLD). The mechanisms of adenovirus-36 (Ad-36) involvement in hepatic steatosis/NAFLD consist in reducing leptin gene expression and insulin sensitivity, augmenting glucose uptake, activating the lipogenic and pro-inflammatory pathways in adipose tissue, and increasing the level of macrophage chemoattractant protein-1, all of these ultimately leading to chronic inflammation and altered lipid metabolism. Moreover, by reducing leptin expression and secretion Ad-36 may have in turn an obesogenic effect through increased food intake or decreased energy expenditure via altered fat metabolism. Finally, Ad-36 is involved in upregulation of cAMP, phosphatidylinositol 3-kinase, and p38 signaling pathways, downregulation of Wnt10b expression, increased expression of CCAAT/enhancer binding protein-beta, and peroxisome proliferator-activated receptor gamma 2 with consequential lipid accumulation.

## 1. Introduction

…*If we want to find out the truth about a certain situation, we have to look at it from a variety of different angles. Furthermore, the best explanation of a phenomenon is achieved by bringing together multiple sources and heterogeneous evidence*…

Obesity represents a major health problem. Epidemiological studies show an increasing prevalence of this condition in every age, gender, race, and socioeconomic group. According to the WHO, in 2016, more than 1.9 billion adults worldwide were overweight including 650 million who were obese. The prevalence of obesity tripled worldwide between 1975 and 2016 [1].

Classical findings demonstrate that, controlling for smoking and reverse causality, obesity is associated with significant increase in all-cause and cause-specific mortality, and in particular with cardiovascular disease mortality [2]. Furthermore, the number of years lived with obesity is directly associated with the risk of mortality [3].

The causes of the obesity pandemic remain unclear. The progressive increase in energy-dense food intake and decrease in physical activity that have occurred in recent years are likely culprits [4], but there is no solid evidence demonstrating their causal role, which has also been contradicted by some experimental results. For instance, an up-to-date nation-wide longitudinal study showed that it is unlikely that the availability of fast food outlets and the lack of physical activity facilities cause obesity in Swedish adults [5]. Alternative putative contributors to the genesis of obesity, worthy of equal consideration, have been proposed. Among them, fresh evidence suggests that microorganisms could have a role in the genesis of obesity [6]. The intriguing but too often overlooked hypothesis of the infective etiology of obesity is the field of investigation of *infectobesity*, a new discipline that assumes that systemic inflammation caused by microorganisms plays a determinant role.

Even though it is well-known that systemic inflammation is associated with obesity, it is still debated as to whether it is a causative factor or a consequence of obesity.

Duncan et al. showed that fibrinogen and other markers of inflammation could predict weight gain in middle-aged adults. In fact, a three-year follow-up of more than 13,000 men and women showed an adjusted odds ratio with regard to a large weight gain (>90th percentile) of 1.65 for those in the highest quartile compared with the lowest quartile of fibrinogen levels [7]. In contrast, recent findings suggest that the inflammatory markers (i.e., hs-C reactive protein (CRP), interleukin (IL)-1Ra, IL-6, tumor necrosis factor (TNF)-alpha and adiponectin), although highly associated with obesity, do not predict—in linear regression models—weight gain in an adult population. This could suggest inflammation as a consequence of obesity rather than a contributing factor to it [8]. One of the first observations that tried to clarify the link between fat depots and chronic inflammation was that adipocytes from both omental and subcutaneous tissue of the obese secrete the pro-inflammatory cytokine IL-6 [9]. The previous finding was challenged by a study showing that, in the presence of abdominal obesity, the contribution of visceral adipose tissue to circulating levels of IL-6 was modest, although IL-6 concentrations are increased in the systemic circulation [10].

It should be stressed that the evidence that chronic inflammation and/or hyperinsulinemia play a casual role in obesity [11] comes mainly from cross-sectional studies, which cannot establish the direction of any causality. Nevertheless, a recent systematic review with meta-analysis showed that chronic inflammation and hyperinsulinemia usually precede changes in higher BMI, which is consistent with a causal role in obesity [12].

This is a key point because it lends credence to a possible factor first inducing chronic inflammation, which in turn leads to the increase in weight.

But which factor could be responsible of this inflammatory status? In the next paragraph we review the evidence suggesting that it could be a viral infection.

## 2. Changing the Scenario: Viruses and Obesity

Six viruses are known to lead to obesity in animals. Canine distemper virus was the first virus reported to cause obesity in mice, followed by Rous-associated virus-7, an avian retrovirus, which has been shown to lead to hyperlipidemia, finite growth, and obesity in chickens. Still, Borna disease virus was found to determine obesity in rats. Small scrapie viruses were revealed to induce obesity in mice and hamsters. Finally, SMAM-1, an avian adenovirus (Ad), and adenovirus-36 (Ad-36), a human adenovirus, were associated with obesity in animals and humans by many researchers [13].

All the obesogenic animal viruses except SMAM-1 cause obesity acting on the CNS. SMAM-1 acts directly on adipocytes and is the only animal virus that is associated with human obesity. Intriguingly, three human adenoviruses, Ad-36, Ad-37, and Ad-5, also directly act on adipocytes. These viruses stimulate enzymes and transcription factors that lead to the storage of triglycerides and help pre-adipocytes differentiate into mature adipocytes. Ad-5 and Ad-37 have also been reckoned to induce obesity in animals. Ad-36 causes obesity in chickens, mice, rats, and monkeys, and, in humans, antibodies against this virus have been found in 30% of obese and 11% of lean individuals [14].

We know that AT (specifically adipocytes) responds to over-nutrition by initiating an inflammatory response [15], but can an inflammatory response be caused by chronic viral infection?

AT serves as a reservoir for the persistence of several viruses including influenza A virus, human immunodeficiency virus (HIV), cytomegalovirus (CMV) and, consistently with its possible role in the genesis of obesity, the human Ad-36 [16,17]. Shifting attention to other viral agents, very recent results suggest that obesity among Qataris may be associated with a higher prevalence of herpesvirus infections, in particular HSV-1. Furthermore, the high prevalence of antibodies against peptide antigens specific to HSV-1 and -2 in the obese population suggests that these viral peptides may play a role in adipogenesis [18].

In addition, the strong pro-inflammatory responses within AT (exerting both autocrine and paracrine effects) and the high number of fat-resident macrophages suggest that AT contributes to anti-infectious innate immune responses [19].

## 3. Prevalence of Adenovirus in the General Population

The prevalence of adenovirus seropositivity differs across ethnic groups ranging from 65% in Italy [20] to 6% in Belgium/Holland [21]. In the Arab population a prevalence of Ad-36 of 47% has been reported: women were more frequently positive and there was no association with normal or increased BMI or the presence of type 2 diabetes mellitus (T2DM) [22].

Among 101 children from elementary schools in southern Brazil, the Ad-36 seropositivity rate was about 16% and was associated with three times higher chance of being overweight. Enrolment in a full-time daycare center before the age of 24 months increased not only the chance of being Ad-36 seropositive, but also of being overweight by little less than three times [23]. Based on these data the question arises whether virus persistence was associated with chronic inflammation and whether adenoviruses are involved.

As mentioned before, in contrast to the acute episode, some viral infections last for long periods, persisting indefinitely within the host because the primary infection is not cleared by the adaptive immune response. Varicella-zoster virus, measles virus, HIV-1, lymphocytic choriomeningitis virus, and human CMV are typical examples of viruses that cause persistent infections [24].

It has long been known that persistent viral infection ends up in a chronic inflammatory status. Intriguingly, during HIV infection, abdominal subcutaneous and visceral adipose tissues are crucial both for continuous viral replication and chronic immune activation and inflammation [25].

Similarly, long-term persistence of infectious Zika virus leads to inflammation and behavioral sequelae in mice [26].

Adenoviruses may cause two types of infection: type 1 or “ineffective”, resulting in transient virus excretion and little or no illness, and type 2 or “persistent”, resulting frequently in symptomatic illness and characterized by long-lasting, intermittent virus excretion. The virus watch program showed that in metropolitan New York families type 2 infection was the most frequent type of adenovirus infection and resulted in prolonged intra-familial spread [27].

Recent results indicate a novel pathogenic role of Th17 cells via interleukin (IL)-17 in persistent viral infection and its associated chronic inflammatory diseases [28]. Of note, serum IL-17 is related to early atherosclerosis in obese patients [29]. IL-17 is produced by helper T (Th) cells that are stimulated by IL-1β and IL-6 derived from phagocytes such as macrophages and from tissue cells/adipocytes. This cytokine strongly contributes to autoimmune diseases that are characterized by chronic inflammation [30].

Furthermore, regulatory B cells (B_regs_) have increasingly gained in attention for restraining inflammation through suppressing the differentiation of Th1 and Th17 immune responses in the development of autoimmune diseases, lending credence to a key player role for IL-17 in these conditions [31].

## 4. Chronic Low-Grade Inflammation, Insulin Resistance and Obesity-Related NAFLD

Metabolic syndrome, of which obesity is an important component has a bidirectional association with nonalcoholic fatty liver disease (NAFLD, recently re-named metabolic associated fatty liver disease (MAFLD), a clinical condition characterized by intra-hepatic fat excess with or without inflammation and fibrosis [32]. NAFLD has the potential of becoming progressive, leading to liver cirrhosis, and is associated with serious cardio-metabolic abnormalities including T2DM [33]

Recent findings demonstrate that insulin resistance in the adipose tissue is prospectively associated with changes in adipose tissue function (adiponectin and soluble CD163), HDL cholesterol, triglycerides, and with incident abnormal glycemia such as the onset of impaired fasting glucose, impaired glucose tolerance, or T2DM at follow-up [34].

Adipose tissue insulin resistance also plays a central role in the development of metabolic and histological abnormalities of obese patients with NAFLD [35]. Intriguingly, accumulating evidence reveals that NAFLD with its related insulin resistance is strongly associated with inflammation, which also has a role in the pathogenesis of one of its more serious complications, the development of hepatocarcinoma (HCC).

More specifically, various pro-inflammatory cytokines (beyond IL-6, TNF-alpha) and adipokines (adiponectin and leptin) play a key role in the onset and worsening of NAFLD-related inflammation by regulating various functions including metabolic energy balance and immune response [36,37].

There is great deal of debate over the need to better distinguish between the two entities of NAFLD (i.e., the simple fatty liver and the more severe form known as nonalcoholic steatohepatitis (NASH)) [38].

In fact, for some authorities, NAFLD encompasses a disease-continuum from steatosis with or without mild inflammation (fatty liver) to NASH, which is characterized by necroinflammation and faster fibrosis progression than fatty liver. Remarkably, NAFLD-related inflammatory status ends up in the emergence of a microenvironment favorable to the development of hepatocarcinoma (HCC) [39]. Indeed, it is speculated that the role of NAFLD in HCC etiology is greater than the data that has been previously reported, and the existence of burned-out NASH is an aspect to be taken into account in epidemiological research dealing with NAFLD-related HCC [40].

HCC is the result of a malignant transformation of normal hepatocytes due to genetic and epigenetic alterations in which inflammation, oxidative stress, and immunity are involved [41].

In fact, non-resolving inflammation is a recognized hallmark of cancer that substantially contributes to the development and progression of HCC. The HCC-associated inflammation can be initiated by extrinsic pathways and propagated through activation of pattern-recognition receptors and by pathogen-associated molecule patterns deriving from gut flora [42].

This key-aspect concerning the gut microbiome will be dealt with later.

Specifically, HCC results in an aberrant activation of different cellular and molecular pathways and disruption of balance between activation and inactivation of protooncogenes and tumor suppressor genes, respectively [43].

A role in HCC pathogenesis has also been proposed for viral infections. Indeed, researchers have described adeno-associated virus type 2 (AAV2) insertional mutagenesis in cancer driver genes in eleven cases of HCC, concluding that AAV2 insertions contribute to a subset of HCC development in rare patients, mainly in the absence of cirrhosis and other risk factors [44].

## 5. Inflammation, Autoimmunity, and Autoantibodies

An intriguing hypothesis that links viral infections to NFLD involves autoimmunity. Increasing pieces of evidence suggest, indeed, that the abnormal inflammatory responses are closely associated with autoimmune diseases [45] and that viral infections are implicated in their onset. Infection triggers a robust and usually well-coordinated immune response that is critical for obtaining viral clearance. However, immune regulatory mechanisms may sometimes halt, culminating in the lack of self-tolerance, which results in immune-mediated attack directed against both viral and self-antigens [46]. There are many examples of viral infections that have been suggested to induce autoimmune diseases including infections due to Coxsackie B virus (CVB), rotavirus, influenza A viruses (IAV), herpesviruses, and measles, mumps and rubella viruses, Zika virus, and Dengue virus [47].

Viruses do not necessarily induce full-fledged autoimmune diseases. Common viral infections are, instead, more often associated with the appearance of low titer, polyspecific autoantibodies [48]. In fact, previous results indicate a relationship between HCV infection and the concurrent detection of various autoantibodies in the absence of symptoms of autoimmune diseases [49]. Anti-nuclear antibodies (ANA) were also found more often in Cytomegalovirus (CMV) antibody-positive sera than in CMV antibody-negative sera, and ANA were usually present in sera that also contained anti-smooth muscle antibodies (ASMA) [50]. Data suggests that Epstein-Barr virus (EBV) could be involved in the ANA and extractable nuclear antigen autoantibody formation [51].

Intriguingly, autoantibodies such as ANA and ASMA may exist in the serum of patients with NAFLD although their prevalence and clinical significance is not clear. Routinely measured autoantibodies have been observed in one quarter of patients with NAFLD and their presence has been associated with more severe histological damage [52]. A study on 864 patients with histology-confirmed NAFLD showed a prevalence of 21% of ANA and/or ASMA at titers respectively ≥ 1:160 and ≥ 1:40 in the absence of autoimmune hepatitis; remarkably, their occurrence was not associated with more advanced histologic features. Authors speculate that autoantibody production in NAFLD subjects may be a consequence of hepatic Natural Killer T cell accumulation [53].

## 6. Adenoviruses and Obesity-Related NAFLD: There Is Much to Discover

The data reviewed in the previous paragraphs suggest a potential link between NAFLD and viral infections via immune-mediated inflammation. How do Ad-36 fit in this scenario? In this paragraph we will show that additional mechanisms unrelated to those described so far could be involved.

Several studies explored Ad seroprevalence in NAFLD. For instance, Trovato et al. showed that Ad-36 seropositive (+) patients had baseline greater BMI with the same level of bright liver score at ultrasound, a feature clearly suggestive of NAFLD but, paradoxically, they showed a better response to intervention (lower salt/lower calories Mediterranean diet, physical activity increase, smoking withdrawal, and lifestyle counselling). Specifically, a more important decrease of BMI, a greater reduction of insulin resistance assessed by HOMA, which is considered as the main driver of NAFLD, and higher prevalence of bright liver disappearance were found [54]. These effects were not dependent on a greater pre-interventional body weight or older age [20]. In the same direction, in another study, the prevalence of anti-Ad-36 antibodies was 32% in an Italian population of NAFLD patients as compared with an infection rate of 47% in those without NAFLD [55].

That said regarding the seroprevalence, some evidence suggests that Adv-36 could affect hepatic glucose metabolism and hepatic glucose output, fatty acid synthesis, and triglyceride formation. In particular, some authors have reported that Adv-36 improved in vivo fasting serum insulin level and HOMA insulin sensitivity in rats [56]. The same group showed that mice experimentally infected with Ad-36 had significantly lower serum levels of several cytokines [57]. These last data could, at least partially, justify the previous mentioned paradoxical findings in humans.

An interesting hypothesis centered on leptin dysregulation could arise from a study in which Ad-36 positivity was determined to be 27.1% and 6% in obese and non-obese children and 17.5% and 4% in obese and non-obese adults, respectively. While there was no difference between Ad-36 positive and negative subjects with regard to total cholesterol, low-density lipoprotein, triglycerides, TNF-alpha, and IL-6 levels, leptin levels were significantly lower in Ad positive subjects [58]. To make matters more complicated, elevated serum leptin seems to be a feature of NAFLD and serum leptin seems to increase as hepatocyte steatosis develops. An enhanced release of leptin is accompanied by a decrease in soluble leptin receptor (sOB-R) concentration, which suggests higher resistance of peripheral tissues toward the action of leptin. The low sOB-R levels in NAFLD patients may be part of a feedback mechanism aimed at counteracting the increase in leptin [59].

A study from Chile confirmed a higher prevalence of Ad-36 positivity in obese than in lean subjects and showed that, in contrast with the canonical finding of hyperleptinemia and leptin resistance in the obese [60], Ad-36 seropositivity was associated with better lipid profile and glycemic control, and lower leptin levels [61].

Whereas increased visceral adipose tissue (VAT) is associated with metabolic dysfunction [62] and is independently associated with NASH or significant liver fibrosis [63], subcutaneous adipose tissue (SAT) increases markers of inflammation and endoplasmic reticulum stress in the liver and both adipose tissue depots [64]. It should be stressed that while adiponectin concentrations were more strongly influenced by VAT, leptin release is dependent on SAT [65].

An intriguing hypothesis emerging from the data reviewed so far is that Ad-36 could be involved in the genesis of hepatic steatosis/NAFLD by reducing leptin gene expression—as feedback mechanism—and insulin sensitivity, by increasing glucose uptake, by activating the lipogenic and pro-inflammatory pathways in adipose tissue, and by increasing the level of macrophage chemo attractant protein-1, ultimately leading to chronic inflammation and altered lipid metabolism [66].

Furthermore, by reducing leptin expression and secretion Ad-36 may have in turn an obesogenic effect through increased food intake or decreased energy expenditure via altered fat metabolism [67].

While by acting on leptin Ad-36 would indirectly impact on NAFLD, these viruses could also directly act on the liver. In this perspective, and in light of the strict link between gut and liver, the so-called liver–gut axis, it is worth to mention that intestinal lymphocytes represent a reservoir for human adenovirus persistence and reactivation, whereas the intestinal epithelium is the main site of viral proliferation preceding dissemination [68].

At this point, we should mention an additional hypothetical mechanism that sound as a paradox. Specifically, Ad-36 markedly decreased serum corticosterone in infected versus control rats [56]. Glucocorticoids exert a diverse array of metabolic functions that have the potential to drive NAFLD, acting on both liver and adipose tissue. In the fasting state, they mobilize lipids from the adipose tissue, increasing fatty acid delivery to the liver, whereas in the fed state, they can promote lipid accumulation [69].

**Figure 1:**
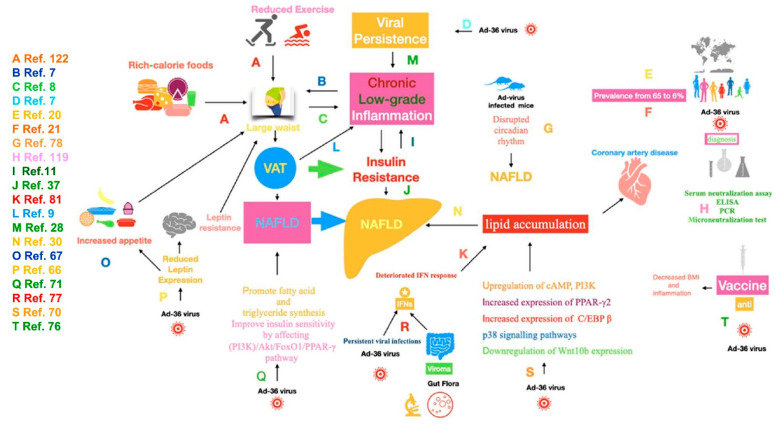
Mechanisms underlying the link between adenovirus-36 and obesity-related NAFLD. NAFLD, nonalcoholic fatty liver disease; VAT, visceral adipose tissue; Ad-36 virus, adenovirus-36; ELISA, enzyme-linked immunosorbent assay; PCR, polymerase chain reaction; PI3K, phosphatidylinositol 3-kinase; AkT, protein kinase B, FoxO1, Forkhead box protein O1; PPAR-γ, peroxisome proliferator-activated receptor gamma; cAMP, cyclic adenosine monophosphate; C/EBP β, CCAAT-enhancer-binding protein beta; p38, p38 mitogen-activated protein kinase. The alphabetic letters indicate the mechanisms likely involved drawn from studies quoted in the related list of references.

Several molecular mechanisms potentially responsible for the “metabolic” effects of AD-36 have been identified. Ad-36 induce the upregulation of cAMP, phosphatidylinositol 3-kinase (PI3K) and p38 signaling pathways, the downregulation of Wnt10b expression, and the increased expression of CCAAT/enhancer binding protein-beta and peroxisome proliferator-activated receptor (PPAR) gamma 2 with consequential lipid accumulation (Figure 1) [70]. Paradoxically, Ad-36 promotes fatty acid and triglycerides synthesis but improves insulin sensitivity by affecting the PI3K/Akt/FoxO1/PPAR-gamma signaling pathway [71].

The E4 open reading frame (orf)-1 (E4ORF1) gene of the virus is necessary and sufficient for Ad-36-induced adipogenesis [70]. In addition, in genetically diabetic (db/db) or diet-induced obesity (DIO) mice, hepatic expression of Ad-36 E4ORF1, but not Ad-5 E4ORF1, greatly improved glycemic control. In normoglycemic wild-type mice, hepatic expression of Ad-36 E4ORF1 lowered non-fasting blood glucose at a high dose of expression. Noteworthy, Ad-36 E4ORF1 significantly reduced insulin levels in db/db and DIO mice. Interestingly, the improvement in glycemic control was observed without stimulation of the proximal insulin signaling pathway [72].

PI3K activation by the adenovirus E4-ORF1 protein, beyond mediating oncogenic cellular transformation (in human Ad-9) and augmenting viral protein expression and replication (in human Ad-5) dysregulates cellular glucose and lipid metabolism (in human Ad-36) by reprogramming it [73].

Ad-36 infection in Wistar rats did not induce any change in inflammation biomarkers including tumor necrosis factor-α, interleukin 6, and monocyte chemoattractant protein-1 levels, but had favorable effects on glycemic and lipid control [74]. These effects may be dependent on E4ORF1, which has been shown to reduce hyperinsulinemia, improve glucose clearance, and protect against hepatic steatosis in younger mice exposed to a high fat diet. Interestingly, E4ORF1, beyond improving glycemic control and liver fat accumulation, maintains mitochondrial integrity and reduces telomere attrition in older mice [75].

Observing the issue from a different point of view, a recent review describes the ongoing development of therapeutic vaccines to treat obesity, and the possibility of using inactivated Ad-36 as a vaccine with anti-obesity properties [76]. This impressive application allows us to consider other factors concerning immunity in our topic.

Interferons (IFNs), cytokines regulating antiviral and autoimmune processes, are crucial in explaining the persistent viral infections that are associated with infectobesity. Furthermore, the IFN-mediated signaling pathway plays a key role in re-programming cellular lipid metabolism, likely clarifying the interaction of viral infection and obesity.

Considering these previous roles, several authors have emphasized the sequential immuno-metabolic adaption to persistent viral infections, pointing out that the deteriorated IFN response, which surfaces as a central mechanism for adipogenesis and obesity-associated immune suppression, could occur because gut microbiota uphold intrinsic IFN signaling [77].

Another interesting hypothesis, adopting genome-wide transcriptomic analyses of intestinal tissues of obese rats with adenovirus infection, revealed signature genes on an inter-systemic scale, many of them involved in the pathways of circadian rhythm, beyond those focusing on IFN signaling as antiviral immunity [78].

There is now an understanding of the complex networking that occurs between the virus and host at the interface of cellular metabolism, with a focus on the IFN-stimulated genes, in particular, cholesterol-25-hydroxylase, spermidine/spermine acetyltransferase 1, indoleamine-2,3-dioxygenase, and a sterile alpha motif as well as histidine/aspartic acid domain-containing protein 1 [79].

## 7. Could IFNs, as Antiviral/Immunomodulant Agents, Play a Role in Obesity-Related NAFLD?

We have seen that, although obesity has various etiologies, an overlooked possibility is obesity of an infectious origin. Accordingly, is there indirect evidence of obesity-related NAFLD as a consequence of a viral disease? Increasing evidence points to IFNs as key players in NAFLD, particularly in the progression to NASH. IFNs’ crucial role in disease development is supported by both genetic evidence and animal studies [80].

Serum concentrations of IFN-α-2, a key cytokine involved in viral infection, were increased in obese subjects with low-prevalence of co-morbidities but with hepatic steatosis [81].

IFN-α-2b treatment protects against diet-induced obesity and alleviates NAFLD in mice [82]. Furthermore, it was shown that both IFN-β1 overexpression and IFN-α-2b administration in mouse models of high fat diet-induced obesity prevent weight gain and suppress immune cell infiltration into AT, attenuate adipose inflammation, and limit AT expansion [83]. Furthermore, obesity-induced expression/responses of type 1-IFN in the liver have been reported to drive the accumulation and activation of intrahepatic CD8+ T cells to promote metabolic syndrome [84]. Treatment with IFN tau, an antiviral agent [85] can significantly mitigate obesity-associated systemic insulin resistance and tissue inflammation by controlling macrophage activity [86]. Furthermore, oral administration of IFN tau enhances oxidation of energy substrates and reduces adiposity in Zucker diabetic fatty rats [87]. It is noteworthy to stress that highlighting the contribution of certain inflammatory responses in obesity or NAFLD-development does not necessarily mean that these inflammatory responses are the result of an infection.

## 8. COVID-19, Obesity and NAFLD

The evidence reviewed so far suggests that viral infection could be responsible for obesity and, possibly, NAFLD. Before further exploring this hypothesis, we have to mention that obesity and NAFLD may, on the other hand, increase the susceptibility to viral infections. This point became dramatically evident during the ongoing COVID-19 pandemic. A recent systematic review showed that individuals with obesity were more at risk for positivity to COVID-19 (OR = 1.46), for hospitalization (OR = 2.13), for intensive care unit admission, (OR = 1.74), and for mortality (OR = 1.48) [88]. Authors found that NAFLD was associated with an increased risk of severe COVID-19, even after adjusting for obesity as a possible confounding factor [89]. NAFLD patients are more likely to show high ALT plasma concentrations when infected by COVID-19 [90]. Additionally, severe COVID-19 cases have been associated with lower expression of IFN-gamma by CD4 T cells [91]. IFN-gamma is an important antiviral protein, and reduced production of this cytokine in response to influenza virus infection has been previously documented in human populations with obesity [92].

Patients may be coinfected by SARS-CoV2, the causative virus of COVID-19, and Ad-36. In fact, a study by Zhu et al. reported 10 out of 257 patients testing positive for co-infection with COVID-19 and adenovirus, the majority of these patients being 15–65 years of age [93].

The interesting consequences of co-infection will be deepened later in a specific paragraph.

## 9. Is There an Involvement of the Spleen?

The innate immune system limits virus replication during systemic infection by producing type I interferons (IFN-I). Nonetheless, viruses may replicate in the spleen despite high levels of IFN-I. In addition, virus specific CD8 + T cells, which are primed in the spleen, migrate to the liver, and kill virus infected cells [94]. Immunity is somehow weak in people with obesity, partially because fat cells infiltrate the organs where immune cells are produced and stored such as the spleen, bone marrow, and thymus. These changes modify the distribution of lymphocyte population, and consequently alter overall immune defense [95]. In mice, high fat diet ingestion caused increased spleen size and CD45+CD3+/CD19+ ratio values in comparison with the control group. Obese mice showed a decrease in NK cell activity and an increase in spleen lymphocyte chemotaxis [96]. It is noteworthy that various cytopathic virus strains were isolated from the splenic lymphocytes of pigs. All viruses were identified as porcine adenoviruses [97].

## 10. What about Gut Virome?

It is well-known that gut microbiome/macrobiome is not limited to bacteria. DNA and RNA viruses that collectively make up the intestinal virome outnumber bacterial cells by as much as 10:1, and include eukaryotic viruses that infect eukaryotic cells, endogenous retroviruses, bacterial viruses (i.e., bacteriophages), and archaeal viruses that infect archaea [98].

According to PCR-based metagenomic studies on fecal shedding, eukaryotic viruses are highly represented in the gut virome of healthy individuals, though with a lower prevalence than phages [99].

For example, viruses from the Anelloviridae, Picobirnaviridae, Adenoviridae, and Astroviridae families and species such as Bocavirus, Rotavirus, Enterovirus, and Sapovirus have been identified in the fecal DNA samples of healthy children [100].

Alterations in the human virome are associated with type-1 diabetes mellitus (T1DM), T2DM, inflammatory bowel disease, HIV infection, and cancer [101]. Interestingly, a significant reduction in the proportion of bacteriophages compared with other intestinal viruses has been observed in patients with advanced NAFLD [102]. Experimental evidence suggests that alterations in gut virome could also have a role in causing obesity. For instance, the transfer of cecal viral communities from mice with a lean phenotype into mice with an obese phenotype led to reduced weight gain and normalized blood glucose parameters—and likely fat depot decrease—relative to lean mice. The authors hypothesized that this effect was mediated via fecal virome transplantation-induced gut microbiome changes [103]. An additional mechanism could involve the interactions between the gut microbiota and B_regs_ considering the relevant role of these lymphocytes in autoimmune diseases and their potential implications in obesity [104].

Accumulating evidence suggests that gut microbiota (possibly also including the gut virome) could play a role in atherosclerosis. Our understanding of the classical risk factors for atherosclerotic onset or progression is rapidly increasing and the complex interactions of inflammation and immune activation in this process have been disclosed. Nevertheless, 30–50% of patients lack typical risk factors, suggesting that other unknown factors, which could include infection or disturbed gut microbiome, are related to the pathogenesis of coronary artery disease [105].

Tang et al. showed that a subset of gut bacteria is involved in the formation of trimethylamine, a metabolite linked to atherosclerosis, and bacteriophages could modulate this process [106].

This observation adds a new perspective to understand the well-known link between NAFLD and ischemic cardiovascular diseases. It has, however, to be underlined that other viruses such as HIV may increase cardiovascular risk by directly or indirectly acting on the vessel wall [107]. Likewise, human CMV and EBV can be found in the arterial wall, which could represent a potential site of persistency of those viruses. The authors also proved a significant association between the presence of human CMV DNA in aortic walls and atherosclerosis. Whether this finding stands for a causal relationship between CMV infection and ischemic heart disease remains unclear [108]. Finally, a significant association was also found between influenza infection and acute myocardial infarction [109].

## 11. Viral Co-Infection: Could That Be the Case with Adenovirus?

The presence of an additional agent may also interfere with the targeted isolation of a known virus. Viral interference, a phenomenon where one virus competitively suppresses replication of other co-infecting viruses, is the most common outcome of viral co-infections [110].

Whatever the mechanism, the sequence of infections, the time interval between one infection and the other, and the route of infection determine whether the outcome of heterologous viral infections will be pathological or protective [110].

Co-infection of RNA viruses is important since it has the potential to enhance viral fitness. Furthermore, through complementation and recombination, co-infection could potentially promote “resurrection” of otherwise defective viral genomes and has the potential to expand viral diversity [111]. Because of the similar modes of transmission of these two viruses, subjects infected with HIV are often co-infected with GBV-C [112], and the the infection persists for decades [113]. Interestingly, 20% of HIV-positive individuals also have hepatitis C [114].

Other examples are Dengue virus–HIV co-infection; HDV–HBV co-infection; Herpes virus–HIV co-infection; Coronavirus–influenza virus; and Human metapneumovirus (HMPV)–respiratory syncytial virus (RSV), the last one resulting in increased disease severity [115].

Could a coinfection with adenoviruses and other viruses such as HCV be involved in the pathogenesis of NAFLD? Even though very little is known on this issue, human adenovirus have the ability to dictate, modulate, and/or alter the course of HCV infection [116].

A recent report showed that immunization of mice with various recombinant adenoviruses, containing antigens from HCV, *Mycobacterium tuberculosis*, HIV, and Ebola virus, all induce robust cross-reactive cellular and humoral immune responses against the HCV antigen cores, NS3 and NS5. The observation of cross-reactive immunity between adenoviruses and HCV is novel and opens new avenues of research in the field [117].

These findings have a special interest in the light of the probable role of HCV in determining NAFLD. HCV genotype 3 has been, indeed, independently associated with hepatic steatosis. Furthermore, superimposed NAFLD and visceral fat are related to lower response rates to antiviral therapy in non-genotype 3 patients. Finally, viral clearance is associated with the resolution of hepatic steatosis in HCV genotype 3, but not other HCV genotypes [118].

## 12. Major Criticisms to the Hypothesis of Ad-36 Involvement in Obesity and NAFLD

Several considerations mitigate the enthusiasm for the viral hypothesis of obesity and NAFLD. Available evidence comes from hardly comparable studies that were performed using different experimental design and laboratory methods. In addition, not all the published studies agreed in supporting the hypothesis and some of them are in contrast with it. Indeed, a very recent systematic review of 37 studies showed a high heterogeneity of diagnostic tests estimating the overall prevalence of Ad-36 infection in a combined population of adults, children, and adolescents. Regarding the diagnostic methods for Ad-36, 21 studies used serum neutralization assay, 12 studies used enzyme-linked immunosorbent assay (one of which used polymerase chain reaction assay (PCR) and serology concomitantly), three studies used PCR as the only diagnostic test, and one study used the microneutralization test for the diagnosis of Ad-36. In addition, strong differences were observed among studies in sample size, which ranged from less than 50 to more than 1000 subjects. No association between Ad-36 infection and obesity was found in four studies and one study showed inverse correlation, in the sense that Ad-36 infection was protective [119].

Another major criticism is that Ad positivity also frequently occurs in lean subjects. How can the presence of Ad-36 infection in non-obese patients be reconciled with a role of these viruses in the genesis of obesity? Interestingly, waist-to-hip ratio was larger in Ad-positive (IgG positive) than in Ad-negative subjects (IgG negative), both in the group of patients with obesity and in lean subjects. This finding could suggest that Ad-virus infection acts as a risk factor for obesity by augmenting abdominal fat depot [120,121]. In other words, Ad-viruses could induce the development of obesity only in the presence of other risk factors.

Finally, it should be emphasized that every association taken into account does not mean causation.

## 13. Conclusions

Canonically, obesity is reckoned to develop not only as a consequence of the imbalance between food intake, mainly rich-calorie food, and scarce caloric utilization, but also of the disequilibrium between white adipose tissue, considered the main site of energy storage, and brown adipose tissue, dedicated to energy expenditure [122]. However, some data demonstrate that a major contributor to obesity—physical inactivity—has not changed substantially at the population level between 1991 and 1998, while the prevalence of obesity (defined as a BMI ≥30 kg/m^2^) increased from 12.0% in 1991 to 17.9% in 1998 [123].

Of the several etiological factors of obesity, infection, an unusual causative/inducing factor, has recently started receiving greater attention. If relevant to humans, infectobesity would be a relatively novel, yet extremely significant, concept [124].

Rationalizing the biological plausibility of infectobesity, Voss and Dhurandhar included direct roles of some viruses to reprogram host metabolism toward a more lipogenic and adipogenic status, the probability that humans may exchange microbiota components—emphasizing its viral component (i.e., virome/virobiota) from livestock reservoirs that have been aggressively selected for efficient weight/fat gain, and finally the adaptation of both the immune and metabolic system of the host under persistent viral infections [125]. The importance of focusing on livestock reservoirs is based on the fact that genomes of these hosts can help identify dominant pathways of virus–host interaction [126].

Although the exact mechanism of pathogen-induced obesity is not completely clear, infection attributable to certain organisms should be included in the long list of potential etiological factors for obesity. Of course, recent progress in immunology could help clarify some mechanisms. For example, Jie et al. found that IL-17 was central to priming hepatic CD8+ T cells since IL-17 neutralization or IL-17RA-knockout ended up in significantly decreased CTL counts and impaired CTL effector functions in adenovirus-infected mice [127].

Clearly, even though evidence that specific viral infections might promote the development of obesity has steadily accumulated, not every case of obesity is of infectious origin [12,128]. Nonetheless, Ad-36 infection could be a potential new factor to be considered to investigate the worldwide epidemic of obesity [129]. Currently, there are no approved therapeutics to treat adenovirus infections, and the standard treatment relies on drugs approved to counteract other viral infections [130].

Now, with NAFLD associated with obesity, this disease could also share, among other causes (i.e., genetic predisposition, obesity, dyslipidemia and T2DM), a viral infection.

The likely mechanisms of Ad-36 involved in NAFLD consist in reducing leptin gene expression and insulin sensitivity, increasing glucose uptake, activating the lipogenic and pro-inflammatory pathways in adipose tissue, leading to chronic inflammation and affecting lipid metabolism. Moreover, Ad-36 reducing leptin expression and secretion may have, in turn, an obesogenic effect through increased food intake or decreased energy expenditure via altered fat metabolism. In light of the strict link between the gut and liver, the authors found that intestinal lymphocytes represent a reservoir for human adenovirus persistence and reactivation, lending credence to a possible impact of adenovirus in the onset or progression of NAFLD.

## Data Availability

Not applicable.

## References

[1] Obesity and Overweight. https://www.who.int/news-room/fact-sheets/detail/obesity-and-overweight.

[2] Lawlor D.A., Hart C.L., Hole D.J., Davey Smith G. (2006). Reverse causality and confounding and the associations of overweight and obesity with mortality. Obesity.

[3] Abdullah A., Wolfe R., Stoelwinder J.U., de Courten M., Stevenson C., Walls H.L., Peeters A. (2011). The number of years lived with obesity and the risk of all-cause and cause-specific mortality. Int. J. Epidemiol..

[4] Hill J.O., Wyatt H.R., Peters J.C. (2012). Energy balance and obesity. Circulation.

[5] Okuyama K., Li X., Abe T., Hamano T., Franks P.W., Nabika T., Sundquist K. (2020). Fast food outlets, physical activity facilities, and obesity among adults: A nationwide longitudinal study from Sweden. Int. J. Obes..

[6] McAllister E.J., Dhurandhar N.V., Keith S.W., Aronne L.J., Barger J., Baskin M., Benca R.M., Biggio J., Boggiano M.M., Eisenmann J.C. (2009). Ten putative contributors to the obesity epidemic. Crit. Rev. Food Sci. Nutr..

[7] Duncan B.B., Schmidt M.I., Chambless L.E., Folsom A.R., Carpenter M., Heiss G. (2000). Fibrinogen, other putative markers of in-flammation, and weight gain in middle-aged adults-The ARIC study. Obes. Res..

[8] Tuomisto K., Jousilahti P., Havulinna A.S., Borodulin K., Männistö S., Salomaa V. (2019). Role of inflammation markers in the prediction of weight gain and development of obesity in adults—A prospective study. Metabol. Open.

[9] Fried S.K., Bunkin D.A., Greenberg A.S. (1998). Omental and subcutaneous adipose tissues of obese subjects release interleukin-6, depot differences and regulation by glucocorticoids. J. Clin. Endocrin. Metab..

[10] Tarantino G., Lobello R., Scopacasa F., Contaldo F., Pasanisi F., Cirillo M., De Caterina M., Conca P., Terracciano D., Gennarelli N. (2007). The contribution of omental adipose tissue to adipokine concentrations in patients with the metabolic syndrome. Clin. Investig. Med..

[11] Erion K.A., Corkey B.E. (2017). Hyperinsulinemia: A Cause of Obesity?. Curr. Obes. Rep..

[12] Wiebe N., Ye F., Crumley E.T., Bello A., Stenvinkel P., Tonelli M. (2021). Temporal Associations Among Body Mass Index, Fast-ing Insulin, and Systemic Inflammation: A Systematic Review and Meta-analysis. JAMA Netw. Open.

[13] Dhurandhar N.V. (2001). Infectobesity: Obesity of infectious origin. J. Nutr..

[14] Atkinson R.L. (2007). Viruses as an etiology of obesity. Mayo Clin. Proc..

[15] Reilly S., Saltiel A. (2017). Adapting to obesity with adipose tissue inflammation. Nat. Rev. Endocrinol..

[16] Dhurandhar N.V. (2011). A framework for identification of infections that contribute to human obesity. Lancet Infect. Dis..

[17] Koethe J.R., McDonnell W., Kennedy A., Abana C.O., Pilkinton M., Setliff I., Georgiev I., Barnett L., Hager C.C., Smith R. (2018). Adipose Tissue is Enriched for Activated and Late-Differentiated CD8+ T Cells and Shows Distinct CD8+ Receptor Usage, Compared With Blood in HIV-Infected Persons. J. Acquir. Immune Defic. Syndr..

[18] Hasan M.R., Rahman M., Khan T., Saeed A., Sundararaju S., Flores A., Hawken P., Rawat A., Elkum N., Hussain K. (2021). Virome-wide serological profiling reveals association of herpesviruses with obesity. Sci. Rep..

[19] Bourgeois C., Gorwood J., Barrail-Tran A., Lagathu C., Capeau J., Desjardins D., Le Grand R., Damouche A., Béréziat V., Lambotte O. (2019). Specific Biological Features of Adipose Tissue, and Their Impact on HIV Persistence. Front. Microbiol..

[20] Trovato G.M., Castro A., Tonzuso A., Garozzo A., Martines G.F., Pirri C., Trovato F., Catalano D. (2009). Human obesity relationship with ad36 adenovirus and insulin resistance. Int. J. Obes..

[21] Goossens V.J., deJager S.A., Grauls G.E., Gielen M., Vlietinck R.F., Derom C.A., Loos R.J., Rensen S.S., Buurman W.A., Greve J.W. (2011). Lack of evidence for the role of human adenovirus-36 in obesity in a european cohort. Obesity.

[22] Lessan N., Saradalekshmi K.R., Alkaf B., Majeed M., Barakat M.T., Lee Z.P.L., Atkinson R.L. (2020). Obesity and Diabetes in an Arab population: Role of Adenovirus 36 Infection. Sci. Rep..

[23] Cancelier A.C.L., V Dhurandhar N., Peddibhotla S., Atkinson R.L., Silva H.C.G., Trevisol D.J., Schuelter-Trevisol F. (2020). Adenovirus 36 infection and daycare starting age are associated with adiposity in children and adolescents. J. Pediatr..

[24] Kane M., Golovkina T. (2010). Common threads in persistent viral infections. J. Virol..

[25] Damouche A., Lazure T., Avettand-Fènoël V., Huot N., Dejucq-Rainsford N., Satie A.P., Mélard A., David L., Gommet C., Ghosn J. (2015). Adipose Tissue Is a Neglected Viral Reservoir and an Inflammatory Site during Chronic HIV and SIV Infection. PLoS Pathog..

[26] Ireland D.D.C., Manangeeswaran M., Lewkowicz A.P., Engel K., Clark S.M., Laniyan A., Sykes J., Lee H.N., McWilliams I.L., Kelley-Baker L. (2020). Long-term persistence of infectious Zika virus: Inflammation and behavioral sequela in mice. PLoS Pathog..

[27] Fox J.P., Brandt C.D., Wassermann F.E., Hall C.E., Spigland I., Kogon A., Elveback L.R. (1969). The virus watch program: A continuing surveillance of viral infections in metropolitan New York families. VI. Observations of adenovirus infections: Virus excretion patterns, antibody response, efficiency of surveillance, patterns of infections, and relation to illness. Am. J. Epidemiol..

[28] Hou W., Kang H.S., Kim B.S. (2009). Th17 cells enhance viral persistence and inhibit T cell cytotoxicity in a model of chronic virus infection. J. Exp. Med..

[29] Tarantino G., Costantini S., Finelli C., Capone F., Guerriero E., La Sala N., Gioia S., Castello G. (2014). Is serum Interleukin-17 associated with early atherosclerosis in obese patients?. J. Transl. Med..

[30] Kuwabara T., Ishikawa F., Kondo M., Kakiuchi T. (2017). The Role of IL-17 and Related Cytokines in Inflammatory Autoimmune Diseases. Mediat. Inflamm..

[31] Duan L., Rao X., Sigdel K.R. (2019). Regulation of Inflammation in Autoimmune Disease. J. Immunol. Res..

[32] Powel E.E., Wai-Sung Wong V., Rinella M. (2021). Non-alcoholic fatty liver disease. Lancet.

[33] Fabbrini E., Sullivan S., Klein S. (2010). Obesity and nonalcoholic fatty liver disease: Biochemical, metabolic, and clinical implications. Hepatology.

[34] Semnani-Azad Z., Connelly P.W., Bazinet R.P., Retnakaran R., Jenkins D.J.A., Harris S.B., Zinman B., Hanley A.J. (2021). Adipose Tissue Insulin Resistance Is Longitudinally Associated With Adipose Tissue Dysfunction, Circulating Lipids, and Dysglycemia: The PROMISE Cohort. Diabetes Care.

[35] Lomonaco R., Ortiz-Lopez C., Orsak B., Webb A., Hardies J., Darland C., Finch J., Gastaldelli A., Harrison S., Tio F. (2012). Effect of adipose tissue insulin resistance on metabolic parameters and liver histology in obese patients with nonalcoholic fatty liver disease. Hepatology.

[36] Tarantino G., Savastano S., Colao A. (2010). Hepatic steatosis, low-grade chronic inflammation and hormone/growth fac-tor/adipokine imbalance. World J. Gastroenterol..

[37] Asrih M., Jornayvaz F.R. (2013). Inflammation as a potential link between nonalcoholic fatty liver disease and insulin re-sistance. J. Endocrinol..

[38] Tarantino G., Conca P., Riccio A., Tarantino M., Di Minno M.N., Chianese D., Pasanisi F., Contaldo F., Scopacasa F., Capone D. (2008). Enhanced serum concentrations of transforming growth factor-beta1 in simple fatty liver: Is it really benign?. J. Transl. Med..

[39] Divella R., Mazzocca A., Daniele A., Sabbà C., Paradiso A. (2019). Obesity, Nonalcoholic Fatty Liver Disease and Adipocyto-kines Network in Promotion of Cancer. Int. J. Biol. Sci..

[40] Ertle J., Dechêne A., Sowa J.P., Penndorf V., Herzer K., Kaiser G., Schlaak J.F., Gerken G., Syn W.K., Canbay A. (2011). Non-alcoholic fatty liver disease progresses to hepatocellular carcinoma in the ab-sence of apparent cirrhosis. Int. J. Cancer.

[41] Yoon K.S. (2018). Molecular mechanisms of hepatocellular carcinoma. Hepatology.

[42] Yu L.X., Ling Y., Wang H.Y. (2018). Role of nonresolving inflammation in hepatocellular carcinoma development and progression. NPJ Precis. Oncol..

[43] Alqahtani A., Khan Z., Alloghbi A., Said Ahmed T.S., Ashraf M., Hammouda D.M. (2019). Hepatocellular Carcinoma: Molecu-lar Mechanisms and Targeted Therapies. Medicina.

[44] Schulze K., Imbeaud S., Letouzé E., Alexandrov L.B., Calderaro J., Rebouissou S., Couchy G., Meiller C., Shinde J., Soysouvanh F. (2015). Exome sequencing of hepatocellular carcinomas identifies new mutational signatures and potential therapeutic targets. Nat. Genet..

[45] Nikoopour E., Schwartz J.A., Singh B. (2008). Therapeutic benefits of regulating inflammation in autoimmunity. Inflamm. Allergy Drug Targets.

[46] Getts D.R., Chastain E.M., Terry R.L., Miller S.D. (2013). Virus infection, antiviral immunity, and autoimmunity. Immunol. Rev..

[47] Smatti M.K., Cyprian F.S., Nasrallah G.K., Al Thani A.A., Almishal R.O., Yassine H.M. (2019). Viruses and Autoimmunity: A Re-view on the Potential Interaction and Molecular Mechanisms. Viruses.

[48] Hansen K.E., Arnason J., Bridges A.J. (1998). Autoantibodies and common viral illnesses. Semin. Arthritis Rheum..

[49] Navarta L.M., Espul C.A., Acosta-Rivero N. (2018). High prevalence of a variety of autoantibodies in a population of hepatitis C virus-infected individuals. APMIS.

[50] Andersen P., Andersen H.K. (1975). Smooth-muscle antibodies and other tissue antibodies in cytomegalovirus infection. Clin. Exp. Immunol..

[51] Cuomo L., Cirone M., Di Gregorio A.O., Vitillo M., Cattivelli M., Magliocca V., Maiorano S., Meledandri M., Scagnolari C., La Rocca S. (2015). Elevated antinuclear antibodies and altered anti-Epstein-Barr virus immune responses. Virus Res..

[52] Adams L.A., Lindor K.D., Angulo P. (2004). The prevalence of autoantibodies and autoimmune hepatitis in patients with nonalcoholic Fatty liver disease. Am. J. Gastroenterol..

[53] Vuppalanchi R., Gould R.J., Wilson L.A., Unalp-Arida A., Cummings O.W., Chalasani N., Kowdley K.V., Nonalcoholic Steatohepatitis Clinical Research Network (NASH CRN) (2012). Clinical significance of serum autoantibodies in patients with NAFLD: Results from the nonalcoholic steatohepatitis clinical research network. Hepatol. Int..

[54] Trovato G.M., Martines G.F., Trovato F.M., Pirri C., Pace P., Garozzo A., Castro A., Catalano D. (2012). Adenovirus-36 Seropositivity Enhances Effects of Nutritional Intervention on Obesity, Bright Liver, and Insulin Resistance. Dig. Dis. Sci..

[55] Trovato G.M., Martines G.F., Garozzo A., Tonzuso A., Timpanaro R., Pirri C., Trovato F.M., Catalano D. (2010). Ad36 adipogenic adenovirus in human non-alcoholic fatty liver disease. Liver Int..

[56] Pasarica M., Shin A.C., Yu M., Ou Yang H.M., Rathod M., Jen K.L., MohanKumar S., MohanKumar P.S., Markward N., Dhurandhar N.V. (2006). Human adenovirus 36 induces adiposity, increases insulin sensitivity, and alters hypothalamic monoamines in rats. Obesity.

[57] Pasarica M., Loiler S., Dhurandhar N.V. (2008). Acute effect of infection by adipogenic human adenovirus Ad36. Arch. Virol..

[58] Karamese M., Altoparlak U., Turgut A., Aydogdu S., Karamese S.A. (2015). The relationship between adenovirus-36 seropositivity, obesity and metabolic profile in Turkish children and adults. Epidemiol. Infect..

[59] Huang X.D., Fan Y., Zhang H., Wang P., Yuan J.P., Li M.J., Zhan X.Y. (2008). Serum leptin and soluble leptin receptor in non-alcoholic fatty liver disease. World J. Gastroenterol..

[60] Considine R.V., Sinha M.K., Heiman M.L., Kriauciunas A., Stephens T.W., Nyce M.R., Ohannesian J.P., Marco C.C., McKee L.J., Bauer T.L. (1996). Serum immunoreactive-leptin concentrations in normal-weight and obese humans. N. Engl. J. Med..

[61] Sapunar J., Fonseca L., Molina V., Ortiz E., Barra M.I., Reimer C., Charles M., Schneider C., Ortiz M., Brito R. (2020). Adenovirus 36 seropositivity is related to obesity risk, glycemic control, and leptin levels in Chilean subjects. Int. J. Obes..

[62] Chau Y.Y., Bandiera R., Serrels A., Chau Y.Y., Bandiera R., Serrels A., Martínez-Estrada O.M., Qing W., Lee M., Slight J. (2014). Visceral and subcutaneous fat have different origins and evidence supports a mesothelial source. Nat. Cell Biol..

[63] Yu S.J., Kim W., Kim D., Yoon J.H., Lee K., Kim J.H., Cho E.J., Lee J.H., Kim H.Y., Kim Y.J. (2015). Visceral Obesity Predicts Significant Fibrosis in Patients with Nonalcoholic Fatty Liver Disease. Medicine.

[64] Gentile C.L., Weir T.L., Cox-York K.A., Wei Y., Wang D., Reese L., Moran G., Estrada A., Mulligan C., Pagliassotti M.J. (2015). The role of visceral and subcutaneous adipose tissue fatty acid composition in liver pathophysiology associated with NAFLD. Adipocyte.

[65] Park K.G., Park K.S., Kim M.J., Kim H.S., Suh Y.S., Ahn J.D., Park K.K., Chang Y.C., Lee I.K. (2004). Relationship between serum adiponectin and leptin concentrations and body fat distribution. Diabetes Res. Clin. Pract..

[66] Sohrab S.S., Kamal M.A., Atkinson R.L., Alawi M.M., Azhar E.I. (2017). Viral Infection and Obesity: Current Status and Future Prospective. Curr. Drug Metab..

[67] Vangipuram S.D., Yu M., Tian J., Stanhope K.L., Pasarica M., Havel P.J., Heydari A.R., Dhurandhar N.V. (2007). Adipogenic human adenovirus-36 reduces leptin expression and secretion and increases glucose uptake by fat cells. Int. J. Obes..

[68] Kosulin K., Geiger E., Vécsei A., Huber W.D., Rauch M., Brenner E., Wrba F., Hammer K., Innerhofer A., Pötschger U. (2016). Persistence and reactivation of human adenoviruses in the gastrointestinal tract. Clin. Microbiol. Infect..

[69] Woods C.P., Hazlehurst J.M., Tomlinson J.W. (2015). Glucocorticoids and non-alcoholic fatty liver disease. J. Steroid Biochem. Mol. Biol..

[70] Rogers P.M., Fusinski K.A., Rathod M.A., Loiler S.A., Pasarica M., Shaw M.K., Kilroy G., Sutton G.M., McAllister E.J., Mashtalir N. (2008). Human adenovirus Ad-36 induces adipogenesis via its E4 orf-1 gene. Int. J. Obes..

[71] Jiao Y., Liang X., Hou J., Aisa Y., Wu H., Zhang Z., Nuermaimaiti N., Zhao Y., Jiang S., Guan Y. (2019). Adenovirus type 36 regulates adipose stem cell differentiation and glucolipid metabolism through the PI3K/Akt/FoxO1/PPARγ signaling pathway. Lipids Health Dis..

[72] McMurphy T.B., Huang W., Xiao R., Liu X., Dhurandhar N.V., Cao L. (2017). Hepatic Expression of Adenovirus 36 E4ORF1 Im-proves Glycemic Control and Promotes Glucose Metabolism Through AKT Activation. Diabetes.

[73] Kumar M., Kong K., Javier R.T. (2014). Hijacking Dlg1 for oncogenic phosphatidylinositol 3-kinase activation in human epi-thelial cells is a conserved mechanism of human adenovirus E4-ORF1 proteins. J. Virol..

[74] Shirani F., Teimoori A., McAinch A.J., Rashno M., Latifi S.M., Karandish M. (2020). Human adenovirus 36 improves insulin sensitivity and lipid profiles and increases inflammatory markers in Wistar rats. J. Investig. Med..

[75] Mostofinejad Z., Akheruzzaman M., Abu Bakkar Siddik M., Patkar P., Dhurandhar N.V., Hegde V. (2021). Antidiabetic E4orf1 protein prevents hepatic steatosis and reduces markers of aging-related cellular damage in high fat fed older mice. BMJ Open Diabetes Res. Care.

[76] Na H.N., Kim H., Nam J.H. (2014). Prophylactic and therapeutic vaccines for obesity. Clin. Exp. Vaccine Res..

[77] Tian Y., Jennings J., Gong Y., Sang Y. (2019). Viral Infections and Interferons in the Development of Obesity. Biomolecules.

[78] Sang Y., Shields L.E., Sang E.R., Si H., Pigg A., Blecha F. (2019). Ileal transcriptome analysis in obese rats induced by high-fat diets and an adenoviral infection. Int. J. Obes..

[79] Raniga K., Liang C. (2018). Interferons: Reprogramming the Metabolic Network against Viral Infection. Viruses.

[80] Møhlenberg M., Terczynska-Dyla E., Thomsen K.L., George J., Eslam M., Grønbæk H., Hartmann R. (2019). The role of IFN in the development of NAFLD and NASH. Cytokine.

[81] Tarantino G., Costantini S., Citro V., Conforti P., Capone F., Sorice A., Capone D. (2019). Interferon-alpha 2 but not Interferon-gamma serum levels are associated with intramuscular fat in obese patients with nonalcoholic fatty liver disease. J. Transl. Med..

[82] Quiroga A.D., Comanzo C.G., Heit Barbini F.J., Lucci A., Vera M.C., Lorenzetti F., Ferretti A.C., Ceballos M.P., Alvarez M.L., Carrillo M.C. (2019). IFN-α-2b treatment protects against diet-induced obesity and alle-viates non-alcoholic fatty liver disease in mice. Toxicol. Appl. Pharm..

[83] Alsaggar M., Mills M., Liu D. (2017). Interferon beta overexpression attenuates adipose tissue inflammation and high-fat diet-induced obesity and maintains glucose homeostasis. Gene.

[84] Ghazarian M., Revelo X.S., Nøhr M.K., Luck H., Zeng K., Lei H., Tsai S., Schroer S.A., Park Y.J., Chng M.H.Y. (2017). Type I Interferon Responses Drive Intrahepatic T cells to Promote Metabolic Syndrome. Sci. Immunol..

[85] Chon T.W., Bixler S. (2010). Interferon-tau: Current applications and potential in antiviral therapy. J. Interferon Cytokine Res..

[86] Ying W., Kanameni S., Chang C.A., Nair V., Safe S., Bazer F.W., Zhou B. (2014). Interferon tau alleviates obesity-induced adipose tissue inflammation and insulin resistance by regulating macrophage polarization. PLoS ONE.

[87] Tekwe C.D., Lei J., Yao K., Rezaei R., Li X., Dahanayaka S., Carroll R.J., Meininger C.J., Bazer F.W., Wu G. (2013). Oral administration of interferon tau enhances oxidation of energy substrates and reduces adiposity in Zucker diabetic fatty rats. Biofactors.

[88] Popkin B.M., Du S., Green W.D., Beck M.A., Algaith T., Herbst C.H., Alsukait R.F., Alluhidan M., Alazemi N., Shekar M. (2020). Individuals with obesity and COVID-19, A global perspective on the epidemiology and biological relationships. Obes. Rev..

[89] Sachdeva S., Khandait H., Kopel J., Aloysius M.M., Desai R., Goyal H. (2020). NAFLD and COVID-19: A Pooled Analysis. SN Compr. Clin. Med..

[90] Huang R., Zhu L., Wang J., Xue L., Liu L., Yan X., Huang S., Li Y., Yan X., Zhang B. (2020). Clinical features of COVID-19 patients with non-alcoholic fatty liver disease. Hepatol. Commun..

[91] Chen G., Wu D., Guo W., Cao Y., Huang D., Wang H., Wang T., Zhang X., Chen H., Yu H. (2020). Clinical and immunological features of severe and moderate coronavirus disease. J. Clin. Investig..

[92] Paich H.A., Sheridan P.A., Handy J., Karlsson E.A., Schultz-Cherry S., Hudgens M.G., Noah T.L., Weir S.S., Beck M.A. (2013). Overweight and obese adult humans have a defective cellular immune re-sponse to pandemic H1N1 influenza A virus. Obesity.

[93] Zhu X., Ge Y., Wu T., Zhao K., Chen Y., Wu B., Zhu F., Zhu B., Cui L. (2020). Co-Infection with respiratory pathogens among COVID-2019 cases. Virus Res..

[94] Lang K.S., Lang P.A. (2015). Balancing viral replication in spleen and liver determines the outcome of systemic virus infection. Z. Gastroenterol..

[95] Kanneganti T.D., Dixit V.D. (2012). Immunological complications of obesity. Nat. Immunol..

[96] Baccan G.C., Hernández O., Díaz L.E., Gheorghe A., Pozo-Rubio T., Marcos A., De La Fuente M. (2013). Changes in lymphocyte subsets and functions in spleen from mice with high fat diet-induced obesity. Proc. Nutr. Soc..

[97] Kadoi K., Iwabuchi M., Satoh T., Katase T., Kawaji T., Morichi T. (1997). Adenovirus isolation from spleen lymphocytes of ap-parently healthy pigs. New Microbiol..

[98] Mukhopadhya I., Segal J.P., Carding S.R., Hart A.L., Hold G.L. (2019). The gut virome: The ‘missing link’ between gut bacteria and host immunity?. Ther. Adv. Gastroenterol..

[99] Minot S., Wu G.D., Lewis J.D., Bushman F.D. (2012). Conservation of gene cassettes among diverse viruses of the human gut. PLoS ONE.

[100] Reyes A., Blanton L.V., Cao S., Zhao G., Manary M., Trehan I., Smith M.I., Wang D., Virgin H.W., Rohwer F. (2015). Gut DNA viromes of Malawian twins discordant for severe acute malnutrition. Proc. Natl. Acad. Sci. USA.

[101] Santiago-Rodriguez T.M., Hollister E.B. (2019). Human Virome and Disease: High-Throughput Sequencing for Virus Discovery, Identification of Phage-Bacteria Dysbiosis and Development of Therapeutic Approaches with Emphasis on the Human Gut. Viruses.

[102] Lang S., Demir M., Martin A., Jiang L., Zhang X., Duan Y., Gao B., Wisplinghoff H., Kasper P., Roderburg C. (2020). Intestinal Virome Signature Associated With Severity of Nonalcoholic Fatty Liver Disease. Gastroenterology.

[103] Rasmussen T.S., Mentzel C.M.J., Kot W., Castro-Mejía J.L., Zuffa S., Swann J.R., Hansen L.H., Vogensen F.K., Hansen A.K., Nielsen D.S. (2020). Faecal virome transplantation decreases symptoms of type 2 diabetes and obesity in a murine model. Gut.

[104] Berthelot J.M., Jamin C., Amrouche K., Le Goff B., Maugars Y., Youinou P. (2013). Regulatory B cells play a key role in immune system balance. Jt. Bone Spine.

[105] Libby P., Ridker P.M., Hansson G.K. (2011). Progress and challenges in translating the biology of atherosclerosis. Nature.

[106] Tang W.H., Hazen S.L. (2014). The contributory role of gut microbiota in cardiovascular disease. J. Clin. Invest..

[107] David M.H., Hornung R., Fichtenbaum C.J. (2002). Ischemic cardiovascular disease in persons with human immunodeficiency virus infection. Clin. Infect. Dis..

[108] Horváth R., Cerný J., Benedík J., Hökl J., Jelínková I., Benedík J. (2000). The possible role of human cytomegalovirus (HCMV) in the origin of atherosclerosis. J. Clin. Virol..

[109] Peretz A., Azrad M., Blum A. (2019). Influenza virus and atherosclerosis. QJM.

[110] Kumar N., Sharma S., Barua S., Tripathi B.N., Rouse B.T. (2018). Virological and Immunological Outcomes of Coinfections. Clin. Microbiol. Rev..

[111] Aguilera E.R., Pfeiffer J.K. (2019). Strength in numbers: Mechanisms of viral co-infection. Virus Res..

[112] George S.L., Varmaz D., Stapleton J.T. (2006). GB Virus C Replicates in Primary T and B Lymphocytes. J. Infect. Dis..

[113] Barnes A., Allen J.B., Klinzman D., Zhang W., Chaloner K., Stapleton J.T. GBV-C persistence does not require CD4+ T cell preservation, and GBV-C viral load (VL) is weakly inversely related to HIV VL. Proceedings of the 4th IAS Conference on HIV Pathogenesis, Treatment and Prevention.

[114] Soriano V., Vispo E., Labarga P., Medrano J., Barreiro P. (2010). Viral hepatitis and HIV co-infection. Antivir. Res..

[115] Debiaggi M., Canducci F., Ceresola E.R., Clementi M. (2012). The role of infections and co-infections with newly identified and emerging respiratory viruses in children. Virol. J..

[116] Singh S., Vedi S., Samrat S.K., Li W., Kumar R., Agrawal B. (2016). Heterologous Immunity between Adenoviruses and Hepatitis C Virus: A New Paradigm in HCV Immunity and Vaccines. PLoS ONE.

[117] Agrawal B., Gupta N., Vedi S., Singh S., Li W., Garg S., Li J., Kumar R. (2019). Heterologous Immunity between Adenoviruses and Hepatitis C Virus (HCV): Recombinant Adenovirus Vaccine Vectors Containing Antigens from Unrelated Pathogens Induce Cross-Reactive Immunity Against HCV Antigens. Cells.

[118] Bondini S., Younossi Z.M. (2006). Non-alcoholic fatty liver disease and hepatitis C infection. Minerva Gastroenterol. Dietol..

[119] Da Silva Fernandes J., Schuelter-Trevisol F., Cancelier A.C.L., Gonçalves e Silva H.C., de Sousa D.G., Atkinson R.L., Trevisol D.J. (2021). Adenovirus 36 prevalence and association with human obesity: A systematic review. Int. J. Obes..

[120] IBil-Lula I., Stąpor S., Krzywonos-Zawadzka A., Woźniak M. (2014). Is there any link between visceral obesity and adenovirus infections in the Polish population?. Adv. Clin. Exp. Med..

[121] So P.W., Herlihy A.H., Bell J.D. (2005). Adiposity induced by adenovirus 5 inoculation. Int. J. Obes..

[122] Gesta S., Tseng Y.H., Kahn C.R. (2007). Developmental origin of fat: Tracking obesity to its source. Cell.

[123] Mokdad A.H., Serdula M.K., Dietz W.H., Bowman B.A., Marks J.S., Koplan J.P. (1999). The spread of the obesity epidemic in the United States, 1991–1998. JAMA.

[124] Pasarica M., Dhurandhar N.V. (2007). Infectobesity: Obesity of infectious origin. Adv. Food Nutr. Res..

[125] Voss J.D., Dhurandhar N.V. (2017). Viral Infections and Obesity. Curr. Obes. Rep..

[126] Cowled C., Wang L.F. (2016). Animal genomics in natural reservoirs of infectious diseases. Rev. Sci. Tech..

[127] Jie Z., Liang Y., Hou L., Dong C., Iwakura Y., Soong L., Cong Y., Sun J. (2014). Intrahepatic innate lymphoid cells secrete IL-17A and IL-17F that are crucial for T cell priming in viral infection. J. Immunol..

[128] Genoni G., Prodam F., Marolda A., Giglione E., Demarchi I., Bellone S., Bona G. (2014). Obesity and infection: Two sides of one coin. Eur. J. Pediatr..

[129] Ponterio E., Gnessi L. (2015). Adenovirus 36 and Obesity: An Overview. Viruses.

[130] Saha B., Parks R.J. (2020). Recent Advances in Novel Antiviral Therapies against Human Adenovirus. Microorganisms.

